# F4/80^+^ Host Macrophages Are a Barrier to Murine Embryonic Stem Cell-Derived Hematopoietic Progenitor Engraftment* In Vivo*

**DOI:** 10.1155/2016/2414906

**Published:** 2016-10-30

**Authors:** Heather L. Thompson, Nico van Rooijen, Bryce T. McLelland, Jennifer O. Manilay

**Affiliations:** ^1^Quantitative and Systems Biology Graduate Group, School of Natural Sciences, University of California-Merced, Merced, CA 95340, USA; ^2^Department of Molecular Cell Biology, Vrije University, Amsterdam, Netherlands; ^3^Molecular and Cell Biology Unit, School of Natural Sciences, University of California-Merced, Merced, CA 95340, USA

## Abstract

Understanding how embryonic stem cells and their derivatives interact with the adult host immune system is critical to developing their therapeutic potential. Murine embryonic stem cell-derived hematopoietic progenitors (ESHPs) were generated via coculture with the bone marrow stromal cell line, OP9, and then transplanted into NOD.SCID.Common Gamma Chain (NSG) knockout mice, which lack B, T, and natural killer cells. Compared to control mice transplanted with adult lineage-negative bone marrow (Lin^−^ BM) progenitors, ESHP-transplanted mice attained a low but significant level of donor hematopoietic chimerism. Based on our previous studies, we hypothesized that macrophages might contribute to the low engraftment of ESHPs* in vivo*. Enlarged spleens were observed in ESHP-transplanted mice and found to contain higher numbers of host F4/80^+^ macrophages compared to BM-transplanted controls.* In vivo* depletion of host macrophages using clodronate-loaded liposomes improved the ESHP-derived hematopoietic chimerism in the spleen but not in the BM. F4/80^+^ macrophages demonstrated a striking propensity to phagocytose ESHP targets* in vitro*. Taken together, these results suggest that macrophages are a barrier to both syngeneic and allogeneic ESHP engraftment* in vivo*.

## 1. Introduction

Pluripotent embryonic stem cells (ESCs) have the potential to differentiate into any cell in the adult body, making them an ideal source of cells for tissue regeneration when other options are absent [1]. Since ESCs display robust teratoma forming potential* in vivo*, differentiated products are thought to be a better option for cellular replacement of diseased or damaged tissues. However, differentiated ESC derivatives are often short-lived and are undetectable after transplantation* in vivo, *leading us to question the developmental compatibility and possible immune rejection of ESC derivatives in the adult host [2–4].

Syngeneic hematopoietic stem cells (HSCs) from the bone marrow (BM) or umbilical cord blood have been used therapeutically to treat blood diseases [3] and allogeneic BM transplantation has been used to induce tolerance to other nonhematopoietic tissues [5]. Embryonic stem cell-derived hematopoietic progenitors (ESHPs), as well as a variety of terminally differentiated hematopoietic cells, can be cultured* in vitro *[6, 7], and ESHPs express markers commonly found on natural adult and embryonic hematopoietic stem and progenitor cell populations [8, 9]. However, ESHPs often fail to engraft at high levels after transplantation* in vivo, *even in immunodeficient mouse models [8, 10–12].

Investigation of the adult host immune response to ESCs and their derivatives has resulted in some controversy [2]. Several groups have described ESHPs as immune-privileged [13, 14], while others have described their ability to induce responses by T cells and natural killer (NK) cells [3, 12, 15–17]. Macrophages have been observed to respond and phagocytose cells of the embryonic inner cell mass, ESCs, and cultured ESC derivatives [18–21]. We previously showed that macrophages from the 129 and Balb/c mouse strains preferentially phagocytosed ESHPs* in vitro *[21]. Here, we extend those findings and present evidence which supports that host macrophages are an innate immune barrier to ESHP engraftment* in vivo*.

## 2. Materials and Methods

### 2.1. ESC Culture and Differentiation

D3 ESC lines (derived from 129 mice, H-2^b^) were purchased from ATCC (Manassas, VA, USA). ESCs were maintained in an undifferentiated state on mitomycin-C treated STO cells (ATCC) in DMEM supplemented with 15% FBS (Atlanta Biologicals, Norcross, GA), 0.15 mM monothioglycerol (Sigma-Aldrich, St. Louis, MO), 1x Penicillin-Streptomycin (Pen/Strep) (Invitrogen, San Diego, CA), and 1000 U/mL Leukemia Inhibitory Factor (LIF). ESCs were passaged every two to three days by trypsinization (0.25% Trypsin-EDTA (Invitrogen)). Prior to differentiation, ESCs were transferred to 0.1% gelatin-coated plates to wean them from the feeder layer in IMDM (Invitrogen) media supplemented as described above. Cells were incubated in a humidified incubator with 5% CO_2_ at 37°C [21, 22].

ESHPs were differentiated from ESCs using coculture on OP9 stromal cell monolayers (ATCC), as published [21]. Briefly, OP9 cells were cultured in alpha-MEM media (Invitrogen) supplemented with 20% FBS and 1x Pen/Strep. One hundred thirty thousand ESCs were plated on OP9 monolayers at 80% confluency in 150 mm^2^ tissue culture dishes in the presence of 5 ng/mL Flt3L and IL-7 (Peprotech, Rocky Hill, NH) in 20 mL of media. At days 4 and 11, 10 mL of media was added with 10 ng/mL Flt3L and IL-7. At day 7, all cells were harvested using cell lifters, filtered through 64 *μ*m nylon mesh (Small Parts, Inc., Miami Lakes, FL), and replated onto fresh monolayers in the presence of 5 ng/mL Flt3L and IL-7.

### 2.2. Isolation of Hematopoietic Progenitors

ESHPs were harvested and digested in Medium 199 (Invitrogen) containing 0.125% w/v Collagenase D and 0.1% v/v DNAse I (both from Roche, South San Francisco, CA), for 60 minutes at 37°C, followed by dissociation by vigorous pipetting. Cells were washed with “M199+ media” containing 2% FBS in Medium 199 and centrifuged at 2000 rpm for 10 minutes. After dissociation, cells were filtered through nylon mesh. The BM from tibiae and femora from adult mice were also collected as described as a source of adult hematopoietic progenitors [21]. To obtain lineage-negative (Lin^−^) cells from the adult BM, cells were stained with a biotinylated anti-lineage (Lin) cocktail (anti-CD3, CD4, CD8, CD11b, CD19, NK1.1, Gr1, and Ter119), and Lin^+^ cells were positively selected from the whole BM using the EasySep™ Biotin Positive Selection Kit (Stem Cell Technologies, Vancouver, Canada) and two rounds of magnetic selection per the manufacturer's instructors. Lin^−^ BM cells were used in phagocytosis assays and transplantation assays.

For sorting ESHPs, cultured cells were harvested and then blocked with 2.4G2 (anti-CD16/CD32) hybridoma supernatant to block Fc receptors. Then, one of two strategies was used for preenrichment and sorting of ESHPs. In the first strategy, all harvested cells were stained with biotinylated anti-lineage (Lin) cocktail (anti-CD3, CD4, CD8, CD11b, CD19, NK1.1, Gr1, and Ter119), PE-anti-CD41 (clone MWReg30, BioLegend), and APC-anti-CD45 (clone 30F11, BioLegend) for 30 minutes and then washed. In a second incubation, the cells were then stained with streptavidin-Pacific Blue (Invitrogen) to develop the anti-lineage cocktail and DAPI (as a viability marker). Sorting was then performed to obtain CD41^+^ CD45^−^ and CD41^+^ CD45^+^ cells together and/or CD41^−^ CD45^+^ cells on a BD FACS Aria II or Aria III flow cytometer in two steps. First, sorting was performed on “Yield” mode, which allowed for rapid enrichment of the target cells, and then the enriched cells were resorted in “Purity” mode, which allows for more stringent cell isolation. In the second strategy, CD41^+^ progenitors were preenriched by staining with CD41-biotin and EasySep-streptavidin coated magnetic beads (Stem Cell Technologies, Inc., Vancouver, Canada). Three rounds of positive selection on the magnet were performed to collect CD41^+^ cells per the manufacturer's instructions, and the final fraction was then costained with streptavidin-PE, APC-anti-CD45, and DAPI as a viability marker. These cells were then sorted on the flow cytometer using “Purity” mode. Sorted progenitors with either strategy were 85–95% pure after sorting (Supplemental Figure  1 in Supplementary Material available online at http://dx.doi.org/10.1155/2016/2414906).

### 2.3. Transplantation

The UC Merced Institutional Animal Care and Use Committee approved all animal procedures. Mice of the 129 (H-2^b^, CD45.2, Stock #002448) and NSG (H-2^g7^, CD45.1, Stock #005557) strains were purchased from The Jackson Laboratory (West Sacramento, CA). Mice were housed in specific pathogen-free conditions with autoclaved food and sterile water. Mice were given 200 rads of irradiation (a sublethal dose in NSG mice [23]) using a ^137^Cs-source (JL Shepherd and Associates, San Fernando, CA). After irradiation, mice received 2 mg/mL neomycin sulfate in their drinking water for 2 weeks (Sigma). One hundred thousand to 5 × 10^5^ ESHPs were administered intravenously via retroorbital injection. Control mice were transplanted with 5 × 10^4^ Lin^−^ BM progenitors or 5–10 × 10^6^ whole adult BM cells.

### 2.4. Flow Cytometry and Histology

Recipients were analyzed at beginning at day 17 after transplantation to assess chimerism in the BM, spleen, and thymus by flow cytometry. Cells were stained at 4°C for 30 minutes in 2.4G2 (anti-CD16/CD32) hybridoma supernatant to block Fc receptors. Cells were then stained with specific antibody cocktails for 30 minutes in a total volume of 100 *μ*L in FACS buffer, using antibodies to the lineage markers CD3 (clone 2C11), CD4 (GK1.5), CD8 (2.43), B220 (RA3-6B2), IgM (RMM-1), Gr-1 (RB6-8C5), CD11b (M1/70), and F4/80 (BM8). Cells were further costained with CD45.1 (A20) and CD45.2 (104) to mark host and donor hematopoietic cells, respectively. Antibodies were purchased from eBioscience or BioLegend (San Diego, CA). DAPI exclusion was used to identify live cells. Cells were analyzed by gating on live, singlet cell populations on BD FACS Aria II or Aria III flow cytometers and data were analyzed using FlowJo software.

For histological analysis, half of the spleens from ESHP and BM recipients were frozen in Tissue-Tek Optimal Cutting Temperature Compound (Sakura Finetek, Inc., Torrance, CA) and the other half was prepared for flow cytometry. Seven *μ*m thick sections were cut using a cryostat, flash fixed in acetone for 10–15 seconds, and followed by fixation in 4% paraformaldehyde for 10 minutes. F4/80 or rat IgG2a isotype control antibodies at 1 : 50 dilution (BioLegend) were used to stain the tissue section. HistoMouse™-SP Broad Spectrum AEC kit (Invitrogen) was used to detect signal.

### 2.5. Phagocytosis Assay

Phagocytosis assays were performed as previously described [21]. Briefly, single cell suspensions were prepared from NSG and 129 spleens. Macrophages were enriched by adherence to plastic tissue culture dishes overnight in a humidified incubator at 37°C with 5% CO_2_. Nonadherent cells were removed by washing the plates with warm 1x PBS. Adherent cells were removed by trypsinization for 5 minutes at 37°C followed by mechanical lifting using a cell lifter. Fifty thousand macrophages were plated per well with 1 × 10^4^ CD41^+^ ESHPs or Lin^−^ BM “target cells” labeled with CFSE (Molecular Probes). To label target cells, ESHPs or Lin^−^ BM cells were washed twice with 1x PBS prewarmed to 37°C and then resuspended at 1 × 10^6^/mL in PBS. One *μ*L/mL of 5 mM CFSE was added to the cells, which were then incubated at 37°C for 10 minutes, and then washed twice with M199+. Macrophages and labeled target cells were cocultured for 3 hours at 37°C. Phagocytosis assay cultures were then harvested and stained with anti-F4/80 APC (BioLegend) and DAPI. Live F4/80^+^ CFSE^+^ cells were quantified by flow cytometry.

### 2.6. Macrophage Depletion* In Vivo*

NSG mice were depleted of macrophages by treatment with clodronate-loaded liposomes (CLL), whereas control mice were treated with phosphate-buffered saline-loaded liposomes (PLL), obtained through ClodLip BV (http://clodronateliposomes.com/) [24]. At day −3, mice were treated with 0.04 mg of liposomes per gram of mouse weight, and on days −1, +5, +10, and +15 mice were treated with 0.02 mg per gram of mouse weight via intraperitoneal injection, with day 0 representing the day that mice received irradiation and hematopoietic transplant. Spleens and BM cells were analyzed by flow cytometry after animals were sacrificed.

### 2.7. Statistics

To determine the group sizes for transplantation, we utilized data from previous studies in which ESHPs were derived* in vitro* and transplanted into similar genetic backgrounds to the NSG mice [8, 11] to estimate the average expected engraftment success rate* in vivo* and the minimum number of animals per group required to achieve meaningful and statistically sound results. Two-tailed unpaired* t*-tests were performed to test differences in the means between groups using Graph Pad Prism (San Diego, CA). Differences were considered statistically significant if *p* < 0.05.

## 3. Results

### 3.1. ESHPs Display Poor Engraftment Ability* In Vivo*

**Figure 1:**
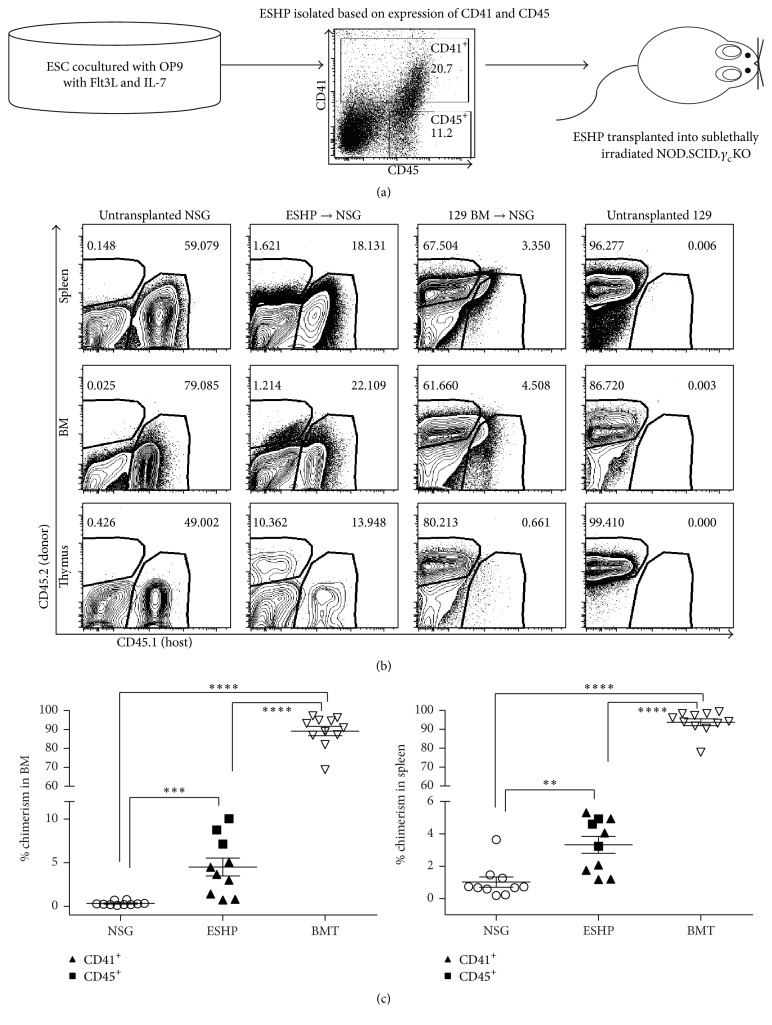
Generation of hematopoietic chimeras using ESHPs. (a) Schematic outline of ESHP generation, isolation, and transplantation. (b) Flow cytometry of hematopoietic chimerism using CD45.2 (donor) on *y*-axis and CD45.1 (host) on *x*-axis in spleen (top), BM (middle), and thymus (bottom). (c) Percent chimerism of total hematopoietic cells in BM (left) and spleen (right) of ESHP recipients (top row) and whole BMT controls (bottom row). Chimerism resulting from transplantation of sorted CD41^+^ ESHP and CD45^+^ ESHP is combined on the graph and for statistical analysis (CD41 filled triangles (*n* = 7), CD45 filled squares (*n* = 3), untransplanted NSG (open circles, *n* = 10), and BMT recipients (open squares, *n* = 11)). Bars represent mean ± SEM; ^*∗∗*^ *p* < 0.01; ^*∗∗∗*^ *p* < 0.001; ^*∗∗∗∗*^ *p* < 0.0001.

Transplantation of* in vitro*-derived ESHPs into adult mouse hosts has not led to high levels of donor chimerism or long-term engraftment without transgenesis [8, 12]. Our previous work [21] suggested that immune responses to ESHPs might be partly responsible for their poor engraftment. To test this further, we utilized mice on the NSG background which lack T, B, and NK lymphocytes but still develop myeloid lineage cells, such as granulocytes, dendritic cells, and, importantly, macrophages [23]. Previous studies have shown the definitive mouse hematopoietic progenitors in the embryo express CD41 and transition through CD41^+^ CD45^−^, then CD41^+^ CD45^+^, and CD41^−^ CD45^+^ stages of maturation [25, 26], and we previously showed that ESHPs with these phenotypes could be generated using a coculture system on the OP9 bone marrow stromal cell line [21]. ESHPs were sorted based on CD41 (least mature) or CD45 (most mature) expression after 16 days of* in vitro* differentiation, as shown in Figure 1(a), in order to compare their levels of engraftment* in vivo*. One hundred thousand to 5 × 10^5^ purified ESHPs were injected into sublethally irradiated NSG hosts. Since 129 and NSG mice differ at the CD45 locus, CD45.1 (expressed by the NSG host strain) and CD45.2 (expressed by 129 donor strain) specific antibodies were used to determine relative levels of donor engraftment (Figure 1(b)). The frequency of host CD45.2^+^ cells was low in ESHP → NSG recipients, as compared to 129 BM → NSG recipients. Controls to distinguish nonspecific “background” staining of anti-CD45.1 versus anti-CD45.2 antibodies were performed using tissues from untransplanted NSG mice (which do not express CD45.2) and untransplanted 129 mice. These results showed that the CD45.2 signal observed in ESHP → NSG recipients was clearly distinguishable from that in untransplanted NSG controls. Donor hematopoietic chimerism averaged 5% or less in the spleen and bone marrow in ESHP recipients, which was low compared to whole adult BMT controls, which averaged about 90% (Figure 1(c)). No significant difference in the level of donor chimerism in recipients of CD41^+^ ESHPs and CD41^−^ CD45^+^ ESHPs was observed. The low level of donor chimerism in ESHP recipients is consistent with the results from other groups [8, 10]. ESHPs were capable of multilineage differentiation, as shown by myeloid differentiation* in vitro* [21] and lymphoid and myeloid differentiation in the bone marrow and spleen* in vivo* (Supplemental Figure 1). Donor chimerism was also evident in the thymus (Figure 1(b)), with signs of T cell development into CD4^+^ CD8^+^ and CD4^+^ CD8^−^ thymocytes in 50% of ESHP recipients (Supplemental Figure 2). Donor chimerism in ESHP recipients was not observed in any tissues after 34 days after transplant. Based on our previous findings [9], we tested the hypothesis that ESHPs were actively rejected by the host innate immune cells.

### 3.2. Host Macrophage Numbers Are Increased in ESHP Recipients

**Figure 2:**
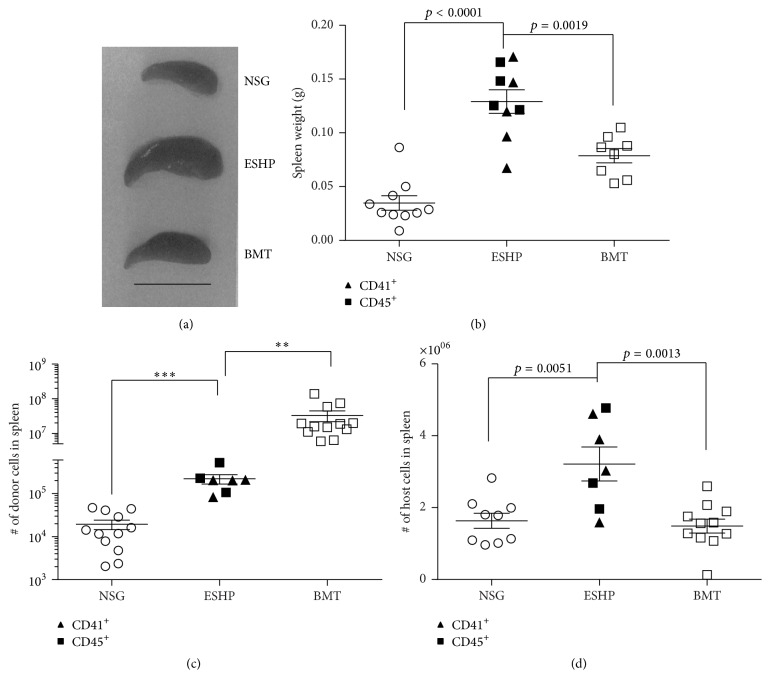
Enlarged spleens in ESHP recipients. (a) Photos of spleens at day 18 after transplant; scale bar = 1 cm. (b) Spleen weights at days 17–34 after transplant for ESHP recipients (CD41 filled triangles (*n* = 5), CD45 filled squares (*n* = 4)), untransplanted NSG (*n* = 10), and whole BMT recipients (*n* = 8). (c) Absolute numbers of donor-derived cells (CD41 (*n* = 4), CD45 (*n* = 3)) and untransplanted NSG (*n* = 11). (d) Absolute numbers of host-derived cells in ESHP recipients (CD41 (*n* = 4), CD45 (*n* = 3)) compared to untransplanted NSG (*n* = 11) controls. Bars represent mean ± SEM; ^*∗∗*^ *p* < 0.01; ^*∗∗∗*^ *p* < 0.001.

Enlarged spleens in ESHP recipients were consistently observed compared to both untransplanted NSG and adult whole BMT controls (Figure 2(a)). To quantify this observation, the spleens were weighed after transplant (Figure 2(b)). The spleens in adult BM-transplanted controls displayed a 2.62-fold increase in mean weight compared to untransplanted NSG mice (Figure 2(b)), consistent with their increased donor hematopoietic chimerism (Figure 1(c)). Similarly, the mean spleen weights in ESHP recipients were increased 3.71-fold compared to untransplanted NSG controls (Figure 2(b)). Although some donor ESHP-derived cells were observed in the spleen (Figure 2(c)), a higher absolute number of host-derived cells in ESHP recipients were present (Figure 2(d)). This number of host-derived cells in the spleen was significantly higher than that of adult BM recipients (Figure 2(d)).

**Figure 3:**
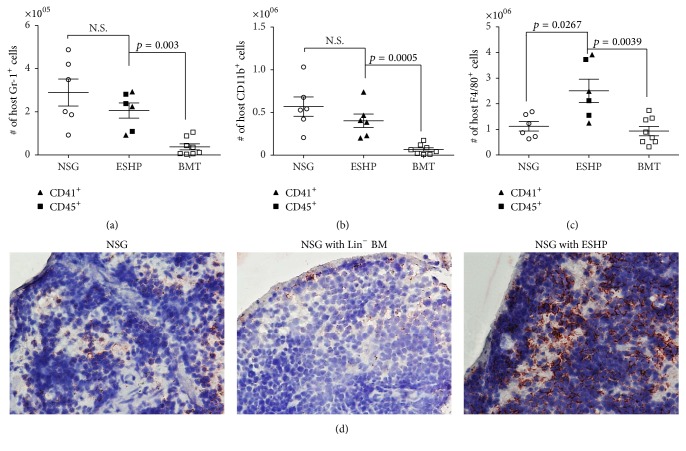
Increase in host F4/80^+^ splenic macrophages in ESHP recipients. (a) Absolute numbers of host-derived Gr-1^+^ cells, (b) absolute numbers of host-derived CD11b^+^ cells, (c) absolute numbers of host F4/80^+^ macrophages in ESHP recipients (CD41 filled triangles, *n* = 3, and CD45 filled squares, *n* = 3), untransplanted NSG mice (*n* = 6), and whole BMT recipients (*n* = 8). Bars represent mean and SEM. (d) Immunohistochemistry of F4/80^+^ macrophages (red-AEC) in spleen sections at days 17–20 after transplant, taken at 40x magnification.

Since NSG mice lack NK, T, and B lymphocytes, we reasoned that only host myeloid cell populations (which include CD11b^+^, Gr-1^+^, and F4/80^+^ macrophages [27] could be increased in the ESHP recipients). Indeed, a significant increase in host-derived CD11b^+^ or Gr-1^+^ cells was observed between ESHP recipients versus adult BMT controls (Figures 3(a) and 3(b)); but the numbers of CD11b^+^ and Gr-1^+^ cells did not account for the observed enlargement of spleen size (Figures 2(a), 3(a), and 3(b)). Remarkably and in contrast, ESHP recipients displayed a statistically significant increase in host F4/80^+^ macrophages compared to both untransplanted NSG and BMT controls (Figure 3(c)), and F4/80^+^ cells were significantly increased in both percentage and absolute number in ESHP → NSG mice compared to 129 BM → NSG (Supplemental Figure 3). The prevalence of F4/80^+^ macrophages was also visible by immunohistochemical staining (Figure 3(d)).

### 3.3. Host Macrophages Preferentially Phagocytose ESHPs* In Vitro*

**Figure 4:**
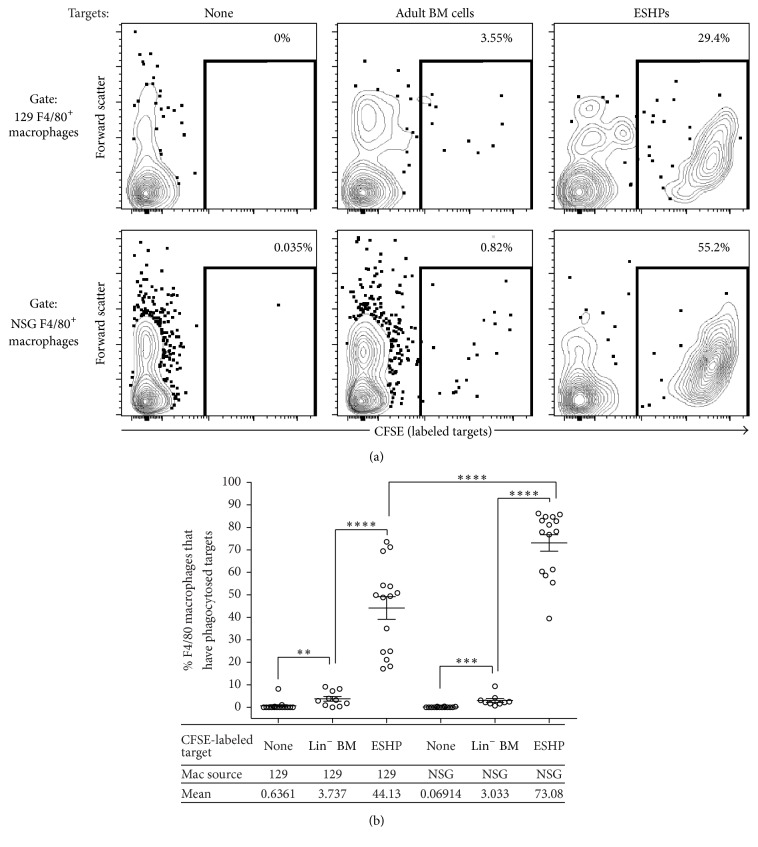
NSG macrophages preferentially phagocytose ESHP* in vitro*. (a) Representative contour plots of CFSE-labeled targets (ESHP or 129 Lin^−^ BM) internalized by NSG F4/80^+^ macrophages* in vitro*. (b) Quantification of targets internalized by F4/80^+^ macrophages. 129 macrophages alone (*n* = 15), 129 macrophages with syngeneic 129 Lin^−^ BM (*n* = 10), 129 macrophages with syngeneic ESHP (*n* = 15), NSG macrophages alone (*n* = 14), NSG macrophages with allogeneic Lin^−^ BM (*n* = 9), and NSG macrophages with allogeneic ESHP (*n* = 15). Bars represent mean ± SEM; ^*∗∗*^ *p* < 0.01; ^*∗∗∗*^ *p* < 0.001; ^*∗∗∗∗*^ *p* < 0.0001.

We hypothesized that ESHPs were actively phagocytosed by host F4/80^+^ macrophages, but the low levels of donor-derived cells in ESHP recipients precluded our ability to test this hypothesis directly* in vivo*. Instead, we used an* in vitro* phagocytosis assay developed in our laboratory, in which phagocytosis of labeled targets can be quantified by flow cytometry [21]. Macrophages from the 129 mouse strain, which is syngeneic to the ESHPs, and macrophages from NSG mice, which are allogeneic to the ESHPs, were used. Regardless of their source, macrophages phagocytosed ESHP targets at a higher rate than control adult Lin^−^ BM targets isolated from the respective strains (11.81- and 24.09-fold higher by syngeneic 129 and allogeneic NSG macrophages, resp. (Figures 4(a) and 4(b))). In addition, NSG macrophages were 1.65-fold more efficient in ESHP phagocytosis than 129 macrophages (Figure 4), suggesting that allogeneic macrophages may react more robustly toward ESHPs than their syngeneic counterparts. These* in vitro* data corroborate the increase in host F4/80^+^ splenic macrophages in the spleens and the low donor hematopoietic chimerism in ESHP recipients observed* in vivo*. Furthermore, flow cytometric measures of forward scatter properties of F4/80^+^ macrophages indicated that host F4/80^+^ macrophages in ESHP → NSG mice were larger in size than F4/80^+^ macrophages in 129 BM → NSG controls, consistent with phagocytosis of ESHPs (Supplementary Figure 4).

### 3.4. Depletion of Macrophages* In Vivo* Increases Donor Hematopoietic Chimerism in ESHP Recipients

**Figure 5:**
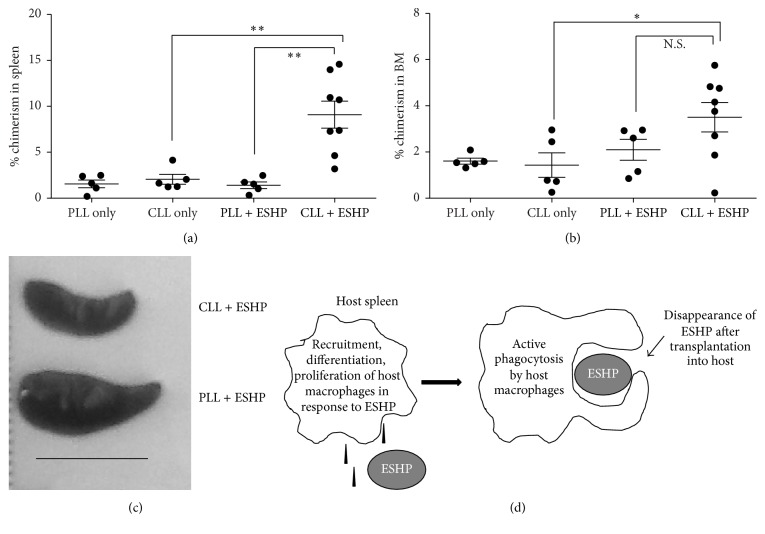
Clodronate depletion of host macrophages increases hematopoietic chimerism in ESHP recipients. (a) Photo of one representative ESHP recipient spleen treated with clodronate-loaded liposomes (CLL) or phosphate-buffered saline-loaded liposomes (PLL); scale bar = 1 cm. (b) Percent donor chimerism in the BM of mice receiving CLL or PLL with or without CD41^+^ ESHP transplant. (c) Percent donor chimerism in the spleens of mice receiving CLL or PLL with or without CD41^+^ ESHP transplant. PLL alone (*n* = 5), CLL alone (*n* = 5), PLL with ESHP (*n* = 5), and CLL with ESHP (*n* = 8). Bars represent mean ± SEM. (d) Model of macrophage ESHP interactions in the spleen. ^*∗*^ *p* < 0.05; ^*∗∗*^ *p* < 0.01.

The increased phagocytosis of ESHPs by allogeneic NSG macrophages provided further support to our hypothesis that macrophages were indeed responsible for poor engraftment of ESHPs after transplantation* in vivo*. To directly test if macrophages were a barrier to ESHP engraftment* in vivo*, clodronate-loaded liposomes (CLL) [24, 28] were used to deplete NSG mice of macrophages prior to and on days 5, 10, and 15 after transplant. CLL treatment specifically depleted F4/80^+^ macrophages subsets in the spleens and BM (Supplemental Figure 5(a)). Donor hematopoietic chimerism was 6.4-fold higher in the spleens of ESHP recipient mice treated with CLL compared to control ESHP-transplanted mice treated with PBS-loaded liposomes (PLL + ESHP) (*p* = 0.0020, Figures 5(a) and 5(b) and Supplementary Figure 5(d)), even though the spleen size of CLL + ESHP-treated mice was smaller (Figure 5(c) and Supplementary Figure 5(c)). In contrast, in the BM, no significant differences in donor chimerism were observed between PLL + ESHP and CLL + ESHP groups (Figure 5(b) and Supplementary Figure 5(E)). CLL treatment did not affect the high level of donor engraftment attained in mice transplanted with whole adult BM cells or enriched Lin^−^ BM progenitors (Supplemental Figure 5(b)).

## 4. Discussion

Our previous work demonstrated indirect recognition of ESHPs by macrophages could stimulate T cell proliferation* in vitro *[21]. In this report, we extend this work to demonstrate that depletion of host macrophages can improve the tissue-specific engraftment of ESHPs* in vivo*. Taken together, we conclude that F4/80^+^ macrophages are a specific immune barrier for ESHPs after transplantation. A working model that summarizes our results is shown in Figure 5(d).

We observed a specific increase in host F4/80^+^ CD11b^−^ macrophages in ESHP recipients and have strong evidence that these macrophages phagocytose ESHPs* in vitro*. Although it is possible that local inflammation in the host could result in the macrophage increases* in vivo*, we have not observed any evidence of contamination in our ESHP cultures or pathogenic infection in our ESHP or control transplanted mice. F4/80^+^CD11b^low/−^ red pulp macrophages have roles in both filtering the blood and removing damaged erythrocytes [29, 30], so we favor the possibility that host red pulp macrophages are induced to expand specifically in response to ESHPs* in vivo*.

The mechanisms that control the phagocytosis of ESHPs are still unknown. We hypothesize that macrophage recruitment and phagocytosis of ESHPs may be related to three aspects of their maturation state: (1) ESHPs secrete products that recruit monocytes and macrophages or induce differentiation or proliferation of macrophages [27], (2) ESHPs express activating ligands [31, 32], and/or (3) ESHPs lack macrophage inhibitory ligands [33–37]. With regard to the latter point, major histocompatibility complex class I (MHC-I) has been characterized as a macrophage inhibitory ligand [29, 38], and ESHPs express MHC-I at moderate levels compared to adult hematopoietic cells [21]. In addition, in preliminary studies, we observed that ESHPs express minimal levels of the macrophage inhibitory ligands CD47 and CD200 (Supplemental Figure 6). Whether induced expression of macrophage inhibitory ligands can improve ESHP engraftment, and whether the host macrophage response is directed to a particular cell subtype within ESHPs, will require further experimentation.

The observation of higher ESHP numbers in the host spleen versus the BM naturally leads to the question of why this is the case. One explanation could be that the spleen is the natural “niche” for ESHPs. In support of this idea, extramedullary hematopoiesis is common in the red pulp region of the spleen in fetal and neonatal mice and decreases in adults as hematopoiesis moves to the BM [39]. Therefore, we speculate that poor engraftment of ESHPs* in vivo *may be caused by a developmental incompatibility between the adult spleen microenvironment and the ESHPs. This is supported by recent evidence that embryonic HSCs demonstrate a propensity to engraft better in neonatal recipients than adult recipients and also populate the niches that best match their developmental stage [40]. Another possibility is that ESHPs may harbor defects in homing that prevents their sufficient migration to the BM cavity and these homing defects reduce their engraftment [10, 41]. Further experimentation to test different routes of ESHP transfer, such as intrafemoral injection [12, 42], is necessary to test this hypothesis. Furthermore, macrophages have been implicated as niche cells that promote retention of HSCs in the BM [43] and our data showed that CLL treatment increased donor hematopoietic chimerism in the spleen but not in the BM of ESHP recipients. If F4/80^+^ host macrophages serve as a BM niche cell population for ESHPs, we posit that their depletion may prevent engraftment of ESHPs in the BM.

There are some caveats of the clodronate liposome system that should be taken into consideration when interpreting our results. Since there is evidence in the literature of toxicity of clodronate liposomes [44] and the liposomes alone can have some nonspecific effect on macrophages [28], we empirically determined the appropriate dosage of PLL and CLL in untransplanted NSG animals to find the optimal dose of liposomes that would deplete the macrophages but not kill the animals. Similar levels of donor chimerism in the ESHP only group and the PLL + ESHP group were expected. However, donor chimerism in the PLL + ESHP group (Figure 5) was observed to be lower than the ESHP only groups (Figure 1(c)), suggesting nonspecific detrimental effects of the control “empty” liposomes on ESHP function. Since we cannot rule out this possibility, we are of the opinion that comparison of donor chimerism in PLL + ESHP versus CLL + ESHP-treated animals is appropriate and better than comparison of donor chimerism in CLL + ESHP-treated mice versus mice that received ESHP without liposomes. We conclude that albeit low, there is clearly a higher level of donor chimerism in the CLL + ESHP-treated mice compared to the PLL + ESHP-treated mice. Furthermore, there is evidence that CLL can deplete cell types other than macrophages, including dendritic cells [45], osteoclasts [46], and neutrophils [47]. Additional studies to target specific subsets of phagocytes are necessary to ascertain their roles on ESHP engraftment.

The derivation and transplantation of ESHPs that resemble adult HSCs are an important goal for the field of hematopoietic stem cell biology. Although transplanting ESHPs using the adult definition of HSC seems straightforward (lineage-negative, cKit^high^, and Sca-1^high^), it is well documented in the literature that embryonic and adult hematopoietic progenitors expressed different surface markers* in vivo* and* in vitro*. In particular, CD41 is expressed on definitive embryonic hematopoietic progenitors* in vivo*, before the expression of the classic CD45 hematopoietic cell marker [25, 26]. Furthermore, McKinney-Freeman et al. subfractionated different ESHP populations from their ESC cultures and found superior engraftment by CD41^+^ ESHPs after transplantation* in vivo *(regardless of cKit or Sca-1 expression) [8] and more recently demonstrated that these cells are not definitive HSCs by transcriptional analysis [48]. In line with these previous studies, our results do not support the assumption that ESHPs share the same markers and behavior as their adult HSC counterparts. We have only observed short-term hematopoietic engraftment with multilineage differentiation from ESHPs transplanted into immunodeficient mice. ESHP engraftment was not expected to reach the same level of BM engraftment, as previous work showed that ESC-derived hematopoietic progenitors with a similar surface phenotype to our ESHPs achieved a wide range of donor chimerism levels in lethally irradiated immunodeficient hosts, but reconstitution was not achieved in 100% of the animals [8]. Since it is technically difficult to obtain sufficient ESHPs in culture for* in vivo* transplantation and there was a high probability of mouse deaths due to lack of reconstitution, we opted to use sublethal irradiation in our studies and optimized the CLL dosage to reduce nonspecific toxicity in the animals. The observation that mice treated with ESHPs do not achieve the same levels of donor chimerism as BMT controls demonstrates that ESHPs may not compete well with host NSG BM progenitors, whereas BM cells from 129 adult donors are able to do so. Treatment with CLL increased the level of donor chimerism in mice that received ESHPs compared to PLL-treated mice. CLL + ESHP-treated mice also showed decreased spleen weights compared to PLL + ESHP-treated mice (Supplemental Figure 3(c)), which could be attributed to the presence of fewer host macrophages in the former, delaying the innate immune rejection of ESHPs. PLL or CLL treatment in BMT recipients did not prevent high levels of engraftment in NSG hosts (data not shown). Taken together, we interpret these data as a demonstration that low engraftment from ESHPs is, in part, due to an innate immune response by host macrophages.

Several groups have reported strong recruitment of macrophages after transplantation of ESC or ESC derivatives undergoing active rejection [19, 20]. Sionov et al. observed that adult macrophages could destroy the inner cell mass (which contain ESCs) from early blastocysts but that the trophoblast repelled these macrophages, suggesting a possible biological mechanism to protect the inner cell mass cells from the maternal (host) immune system macrophages during early embryonic development [18]. It is possible that this natural embryonic trophoblast mechanism, which prevents adult macrophages from rejecting embryonic cells, might be leveraged to protect* in vitro* ESC-derived tissues after transplantation in adult hosts. Current treatments for hemophagocytic lymphohistiocytosis and macrophage activation syndromes include broad immunosuppressive therapy [49–51] which could accompany any ESHP transplantation in humans. Recently, the janus kinase inhibitor ruxolitinib was shown to improve the symptoms of HLH in murine models [49] and this is another possible direction for future human ESHP transplants. Strategies to skew polarization of macrophages toward proinflammatory M1 versus regulatory M2 phenotypes are currently being explored for clinical applications (although further studies are required to determine the M1 versus M2 phenotype of the F4/80^+^ macrophages in the ESHP → NSG chimeras) [52]. To our knowledge, a comparison of the expression levels and functional abilities of macrophage inhibitory ligands and in the NSG versus 129 strains has not been performed. However, there is evidence of mouse strain differences in the binding of the ability of the NOD, BALB/s, and B6 forms of SIRP*α* to bind to the human macrophage inhibitor receptor CD47 and prevent phagocytosis of xenogeneic cells in mice [53]. Our observations that ESHPs are phagocytosed by host macrophages* in vitro* and that clodronate treatment promotes higher donor chimerism from ESHPs in mice* in vivo* strongly suggest that ESHPs stimulate innate immune responses and that control of macrophage-induced immune rejection should be considered in the field as new hematopoietic derivatives are produced from ESCs or ESC-like induced pluripotent stem cells for* in vivo *transplantation.

## Supplementary Material

Supplementary Figure 1: ESHPs are capable of multi-lineage development *in vivo*.
Supplementary Figure 2: ESHPs reconstitute the thymus of NSG mice.
Supplementary Figure 3: Percentages of host myeloid cells in the spleen of chimeras.
Supplementary Figure 4: Increased size of F4/80+ macrophages in ES-HP recipients.
Supplementary Figure 5: Clodronate loaded liposomes (CLL) specifically deplete F4/80+ macrophages but do not affect donor chimerism levels in BM-transplanted mice.
Supplementary Figure 6: Expression of macrophage inhibitory ligands CD47 and CD200 on ESHP.

## Abbreviations

BM: Bone marrow
BMT: BM-transplanted
CLL: Clodronate-loaded liposomes
ESC: Embryonic stem cell
ESHP: Embryonic stem cell-derived hematopoietic progenitor
HSC: Hematopoietic stem cell
Lin: Lineage
NSG: NOD-*scid* IL2R*γ*-null mouse
PLL: Phosphate-buffered saline-loaded liposomes.
