# Supplementary material for: F4/80+ Host Macrophages Are a Barrier to Murine Embryonic Stem Cell-Derived Hematopoietic Progenitor Engraftment In Vivo
Source: J Immunol Res. 2016 Oct 30;2016:2414906. doi: 10.1155/2016/2414906 (PMC5107259; doi:10.1155/2016/2414906)
Supplement: Supplementary file 1 — Supplementary Figure 1: ESHPs are capable of multi-lineage development in vivo. Supplementary Figure 2: ESHPs reconstitute the thymus of NSG mice. Supplementary Figure 3: Percentages of host myeloid cells in the spleen of chimeras. Supplementary Figure 4: Increased size of F4/80+ macrophages in ES-HP recipients. Supplementary Figure 5: Clodronate loaded liposomes (CLL) specifically deplete F4/80+ macrophages but do not affect donor chimerism levels in BM-transplanted mice. Supplementary Figure 6: Expression of macrophage inhibitory ligands CD47 and CD200 on ESHP. [file 2414906.f1.pdf]

Supplementary Figure 1: ESHPs are capable of multi-lineage development *in vivo.*

A. Data are shown from the bone marrow and spleens of from NSG (CD45.1+) hosts transplanted with donor (CD45.2+) ESHPs (left column) or adult bone marrow cells (middle columns). Cell staining from a wild-type 129 (CD45.2+) mouse is shown for comparison (right columns). The FACS data in the second row is gated on live, donor –derived (CD45.2+) cells and shows myeloid lineages.

B. Donor-derived B cell populations in the bone marrow and spleen of recipients of ESHP, (left) BM cells (middle) and a untransplanted control. Data in the top row are gated on live, donor –derived (CD45.2+) cells. The bottom row shows IgM expression on B220+CD19+ B cell populations (black lines) and B220-CD19- non-B cells (filled gray histogram).

C. Example of purity of CD41+ and CD45+ ESHPs after flow cytometric sorting. Cells were gated based on forward scatter and side scatter properties, singlets, and viable cells based on DAPI exclusion.

Figure S1

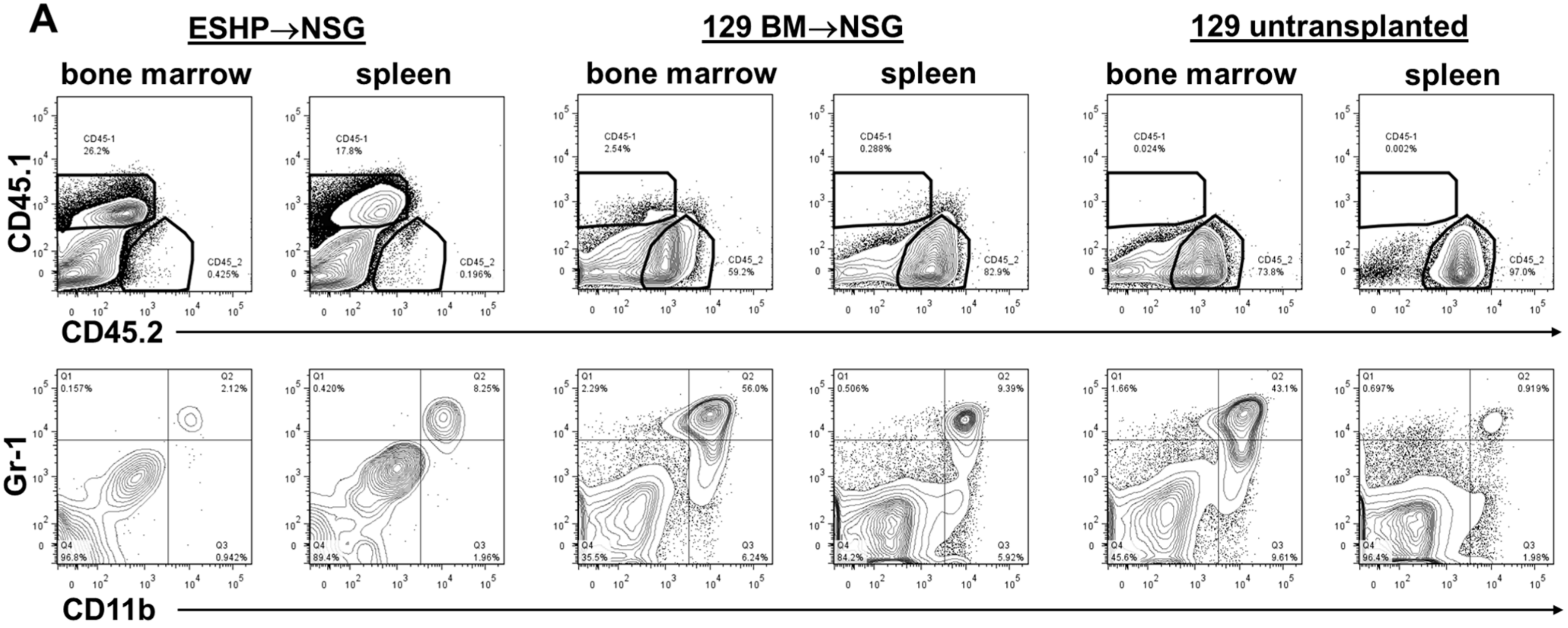
A
ESHP→NSG
129 BM→NSG
129 untransplanted
bone marrow
spleen
bone marrow
spleen
bone marrow
spleen
CD45.1
CD45.2
Gr-1
CD11b
CD45-1
26.2%
CD45_2
0.425%
CD45-1
17.8%
CD45_2
0.196%
CD45-1
2.54%
CD45_2
59.2%
CD45-1
0.288%
CD45_2
82.9%
CD45-1
0.024%
CD45_2
73.8%
CD45-1
0.002%
CD45_2
97.0%
Q1 0.157%
Q2 2.12%
Q3 0.942%
Q4 96.8%
Q1 0.420%
Q2 8.25%
Q3 1.96%
Q4 89.4%
Q1 2.29%
Q2 56.0%
Q3 6.24%
Q4 35.5%
Q1 0.506%
Q2 9.39%
Q3 5.92%
Q4 84.2%
Q1 1.66%
Q2 43.1%
Q3 9.61%
Q4 45.6%
Q1 0.697%
Q2 0.919%
Q3 1.98%
Q4 96.4%

Figure S1

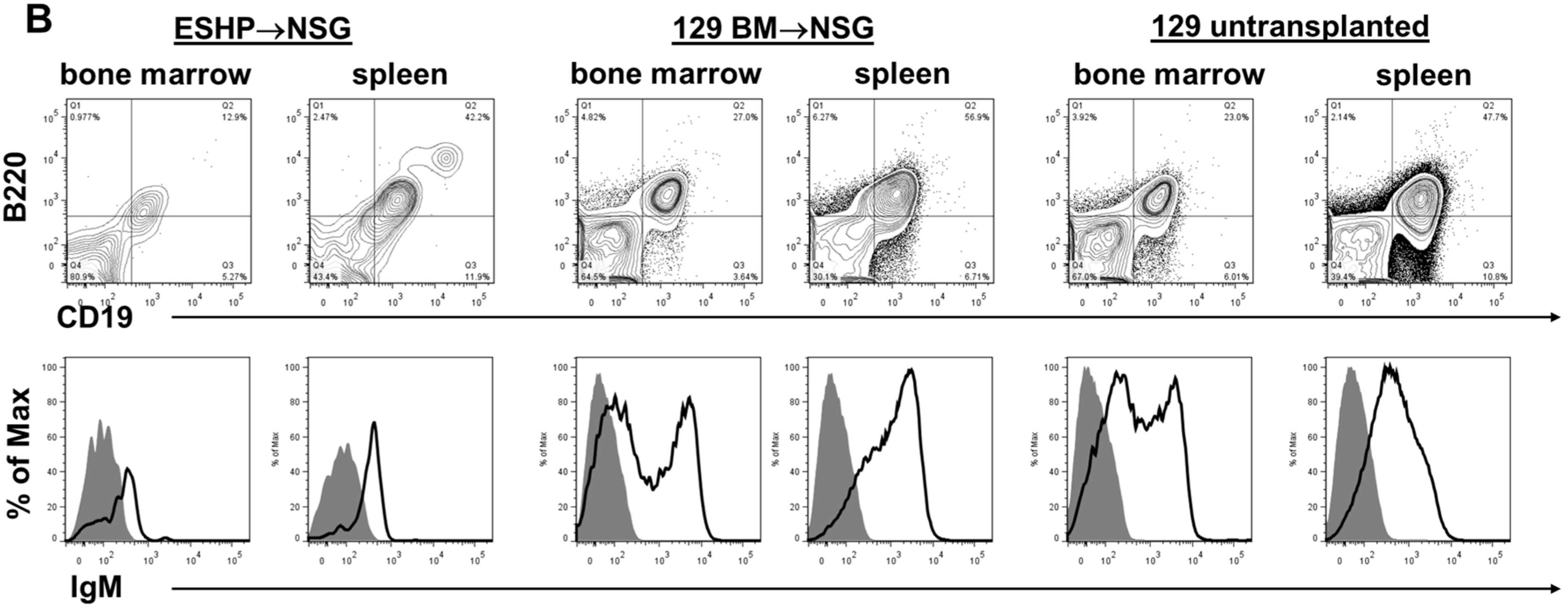

B
ESHP→NSG
129 BM→NSG
129 untransplanted
bone marrow
spleen
bone marrow
spleen
bone marrow
spleen
B220
CD19
% of Max
IgM
Q1 0.977%
Q2 12.9%
Q3 5.27%
Q4 80.9%
Q1 2.47%
Q2 42.2%
Q3 11.9%
Q4 43.4%
Q1 4.82%
Q2 27.0%
Q3 3.64%
Q4 64.5%
Q1 6.27%
Q2 56.9%
Q3 6.71%
Q4 30.1%
Q1 3.92%
Q2 23.0%
Q3 6.01%
Q4 67.0%
Q1 2.14%
Q2 47.7%
Q3 10.8%
Q4 39.4%

Figure S1

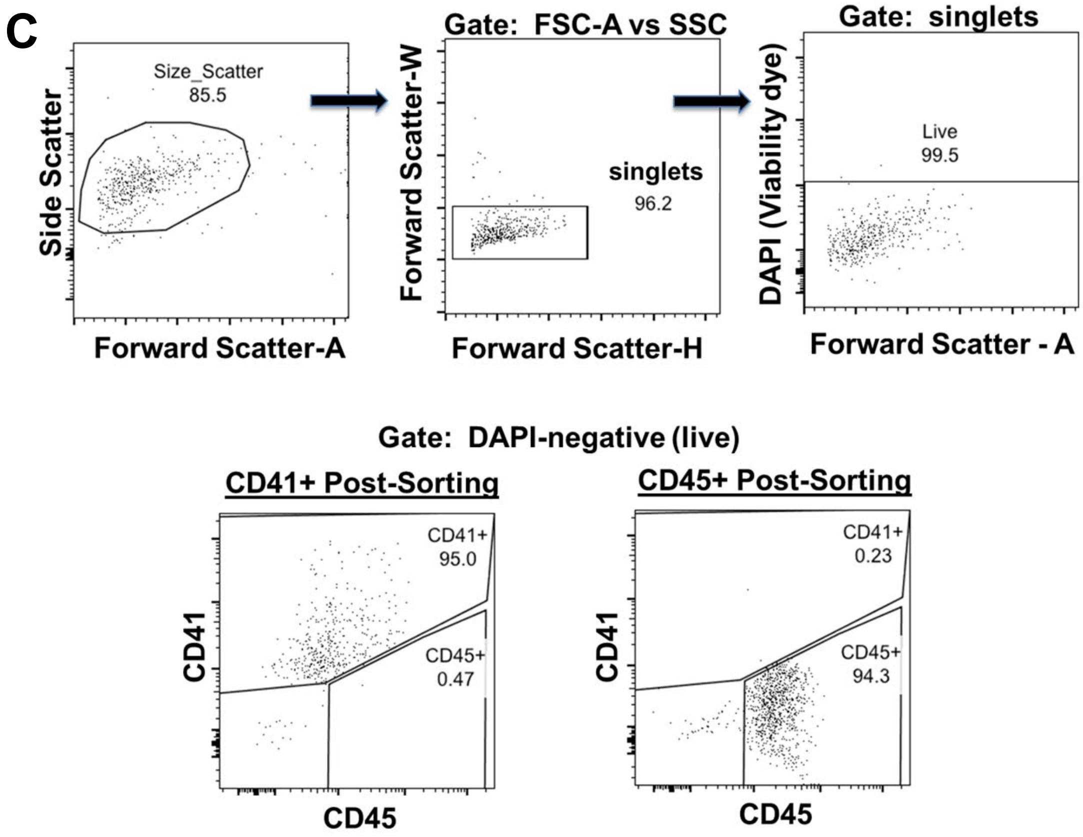

C
Size_Scatter
85.5
Side Scatter
Forward Scatter-A
Gate: FSC-A vs SSC
Forward Scatter-W
singlets
96.2
Forward Scatter-H
Gate: singlets
DAPI (Viability dye)
Live
99.5
Forward Scatter - A
Gate: DAPI-negative (live)
CD41+ Post-Sorting
CD41+
95.0
CD45+
0.47
CD41
CD45
CD45+ Post-Sorting
CD41+
0.23
CD45+
94.3
CD41
CD45

Supplementary Figure 2: ESHPs reconstitute the thymus of NSG mice. Data are shown from the thymus of untransplanted NSG (CD45.1+) (top row), NSG transplanted with ESHPs second row), NSG transplanted with adult bone marrow cells (third row), and an untransplanted 129 mouse (CD45.2+). The untransplanted NSG and 129 samples were used as a specificity control for anti-CD45.1 and anti-CD45.2 staining. The left column shows hematopoietic chimerism using antibodies for specific host CD45.1 and donor CD45.2 expression. The middle column is gated on CD45.2 (donor) cells with staining for T cell populations. The right column shows CD3 staining on CD4+ single positive T cells (Blue), CD4+ CD8+ double positive T cells (gray), and CD8+ single positive cells (Black).

Figure S2

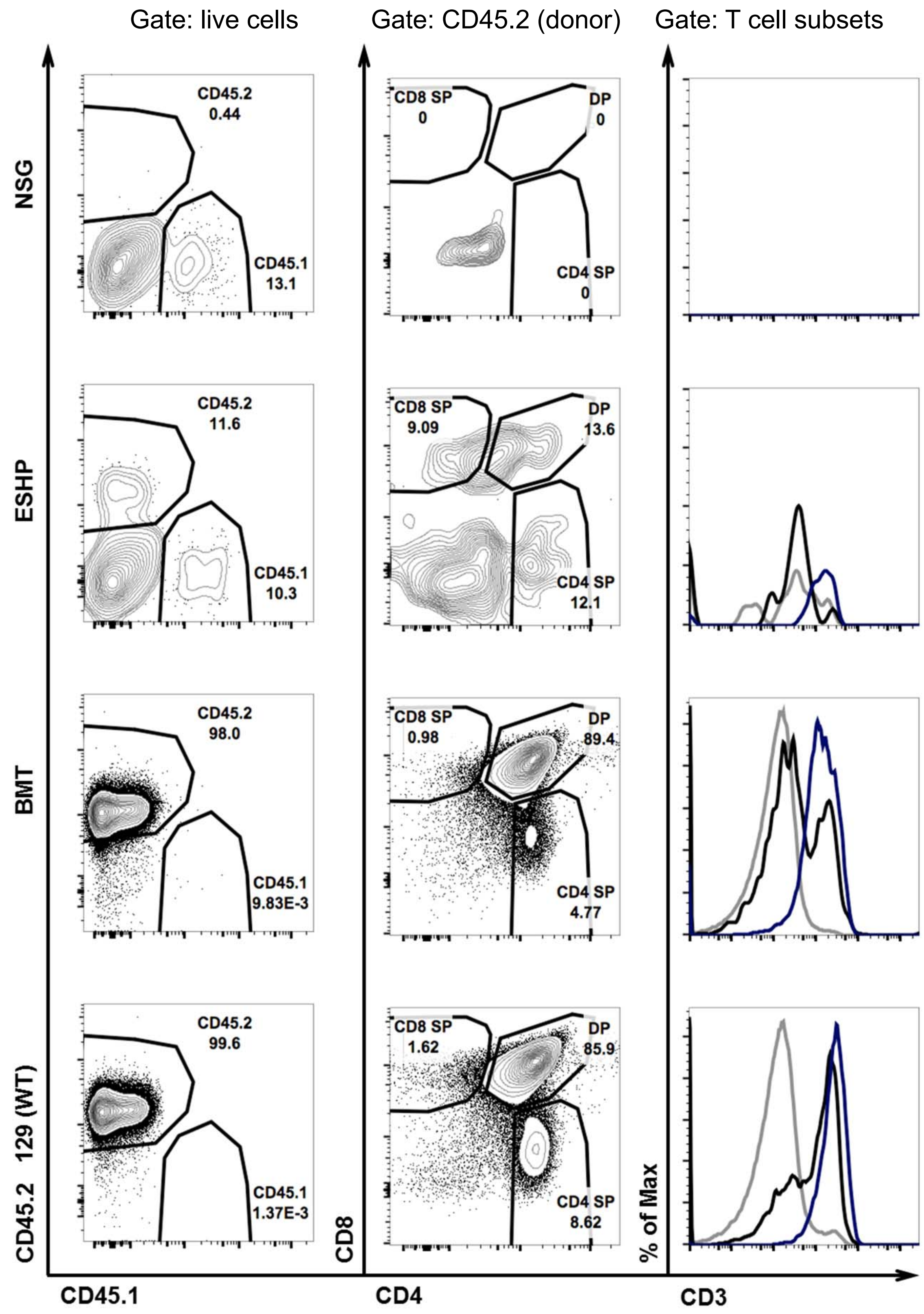

Gate: live cells
Gate: CD45.2 (donor)
Gate: T cell subsets
NSG
CD45.2
0.44
CD45.1
13.1
CD8 SP
0
DP
0
CD4 SP
0
ESHP
CD45.2
11.6
CD45.1
10.3
CD8 SP
9.09
DP
13.6
CD4 SP
12.1
BMT
CD45.2
98.0
CD45.1
9.83E-3
CD8 SP
0.98
DP
89.4
CD4 SP
4.77
CD45.2 129 (WT)
CD45.2
99.6
CD45.1
1.37E-3
CD8 SP
1.62
DP
85.9
CD4 SP
8.62
CD45.1
CD8
CD4
% of Max
CD3

Supplemental Figure 3: Percentages of host myeloid cells in the spleen of chimeras. A) % of host Gr1+ cells; B) % of host CD11b+ cells; and C) % of host F4/80+ cells. Data from untransplanted, non-irradiated NSG, ESHP$\rightarrow$NSG and 129 BM $\rightarrow$ NSG recipients are shown.

.

Figure S3

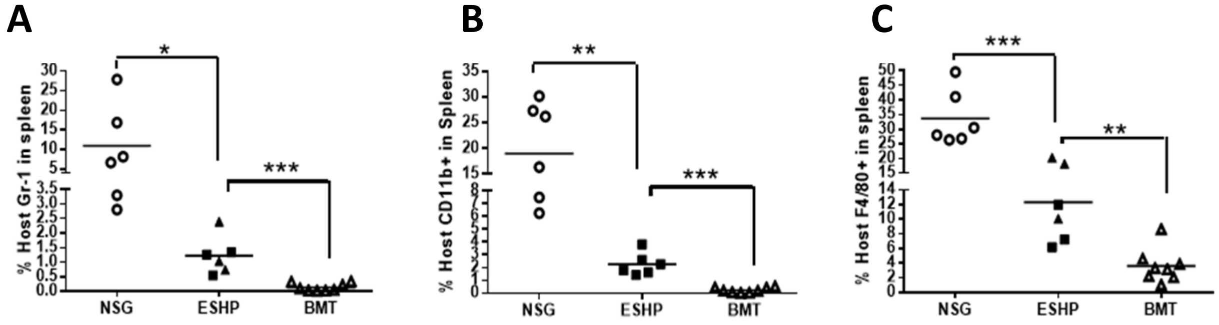

Mean % ± SD	**Untransplanted NSG**	**ESHP→ NSG**	**129 BM → NSG**
**Host Gr1+**	10.94 ± 9.724	1.22 ± 0.6497	0.1364 ± 0.1369
**Host CD11b+**	18.98 ± 10.49	2.25 ± 0.8781	0.2418 ± 0.2025
**Host F4/80+**	33.73 ± 9.455	12.30 ± 5.778	3.592 ± 2.314

Supplemental Figure 4: Increased size of F4/80+ macrophages in ES-HP recipients.

A. Representative histogram of FSC-A on host F4/80+ macrophages. Heavy black line, ESHP-> NSG; heavy dark grey line, 129 BM -> NSG, and light gray tinted region; untransplanted NSG macrophages.

B. Mean fluorescence intensity (MFI) for forward scatter area (FSC-A) of host F4/80+ cells in untransplanted NSG, ESHP -> NSG, and 129 BM -> NSG (BMT) spleens. Statistical signfiicance was measured using Student's t-test.

Figure S4

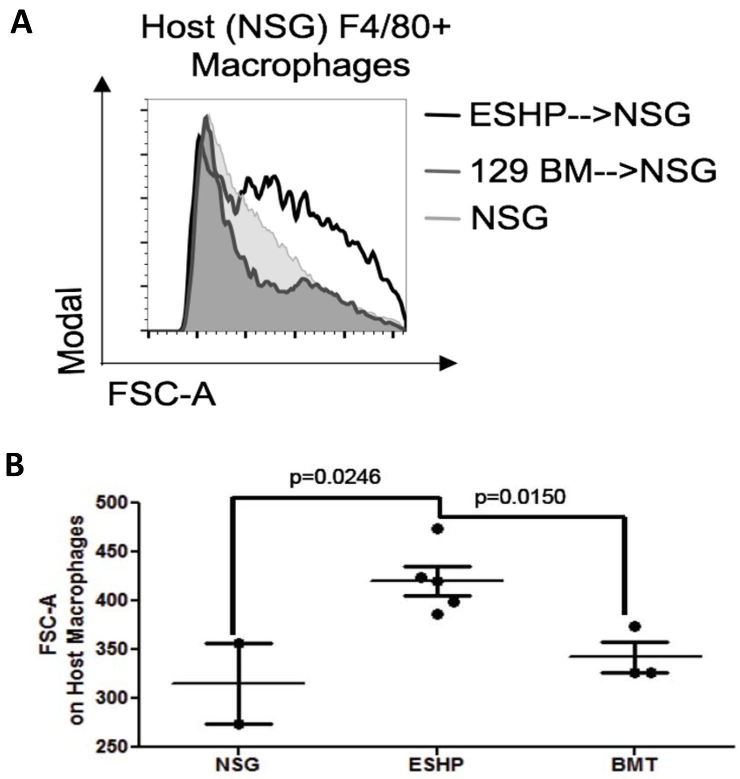
A
Host (NSG) F4/80+
Macrophages
ESHP-->NSG
129 BM-->NSG
NSG
Modal
FSC-A
B
p=0.0246
p=0.0150
FSC-A
on Host Macrophages
500
450
400
350
300
250
NSG
ESHP
BMT

Supplemental Figure 5: Clodronate loaded liposomes (CLL) specifically deplete F4/80+ macrophages but do not affect donor chimerism levels in BM-transplanted mice.

A. A dose titration of CLL was performed to find the optimal dose for macrophage depletion with the least toxicity. Mice were administered CLL at 0.02, 0.01, 0.05 mg/g mouse weight. The FCM plots show the profiles of the remaining macrophages in the spleen and BM of 0.02 mg/g CLL and PLL-treated animals. B. % donor chimerism in the spleens (left) and bone marrow (BM, right) in mice receiving PLL or CLL treatment, with either transplantation of Lin- BM or whole BM cells. C. Spleen weights in CLL and PLL treated mice after transplantation. Statistical significance was measured using Student's t-test. D. Representative flow cytometry plots showing donor and host-derived cells in the spleens of mice receiving CLL only, CLL + ESHP, PLL only, or PLL+ESHP, along with histograms of spleens from untransplanted 129 and NSG mouse strains to demonstrate anti-CD45.2 and anti-CD45.1 antibody specificity. E. Similar to D, but for bone marrow samples.

Figure S5

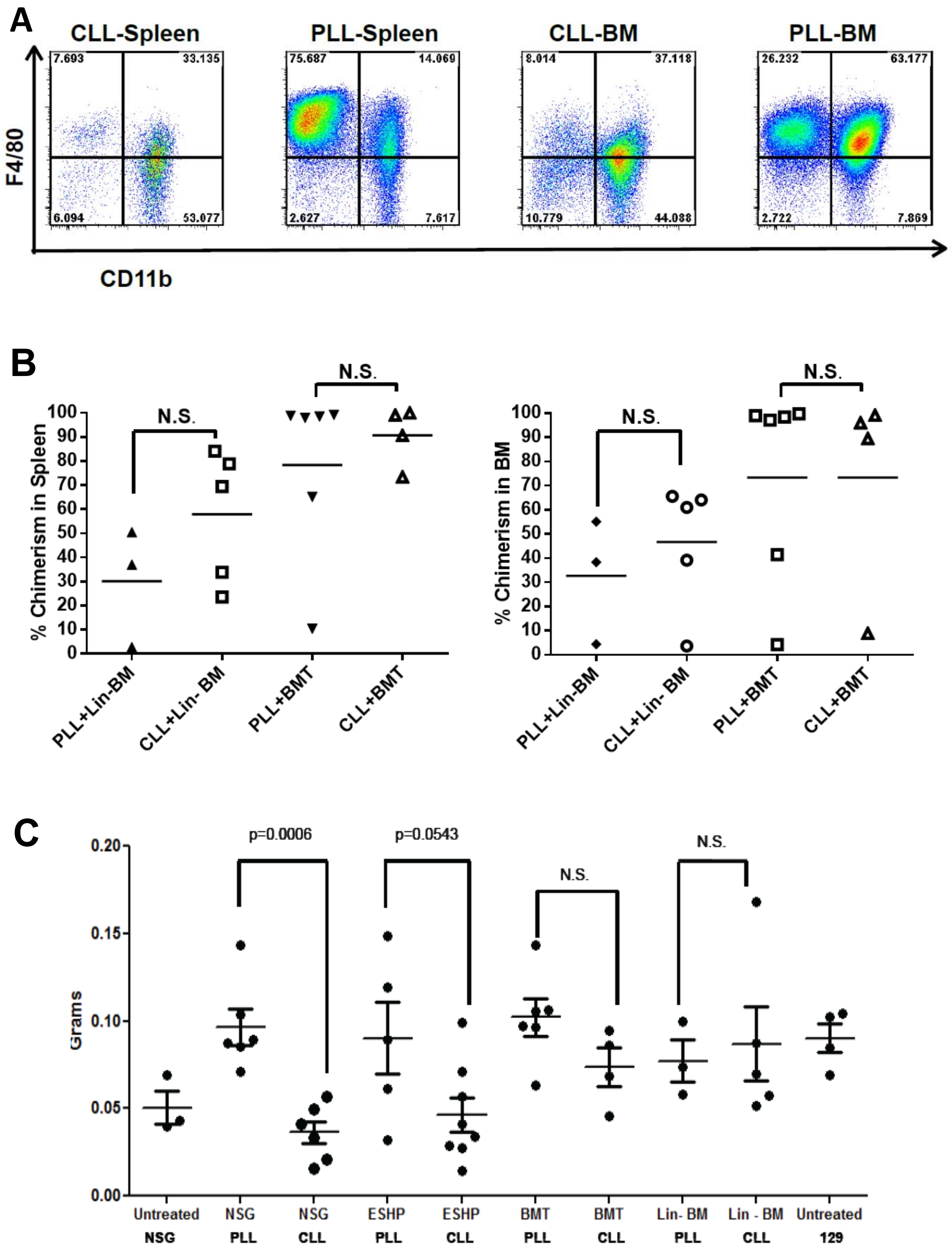

A
CLL-Spleen
PLL-Spleen
CLL-BM
PLL-BM
F4/80
CD11b
7.693
33.135
6.094
53.077
75.687
14.069
2.627
7.617
8.014
37.118
10.779
44.088
26.232
63.177
2.722
7.869
B
N.S.
% Chimerism in Spleen
% Chimerism in BM
PLL+Lin-BM
CLL+Lin- BM
PLL+BMT
CLL+BMT
C
p=0.0006
p=0.0543
Grams
Untreated NSG
NSG PLL
NSG CLL
ESHP PLL
ESHP CLL
BMT PLL
BMT CLL
Lin- BM PLL
Lin - BM CLL
Untreated 129

Figure S5

D

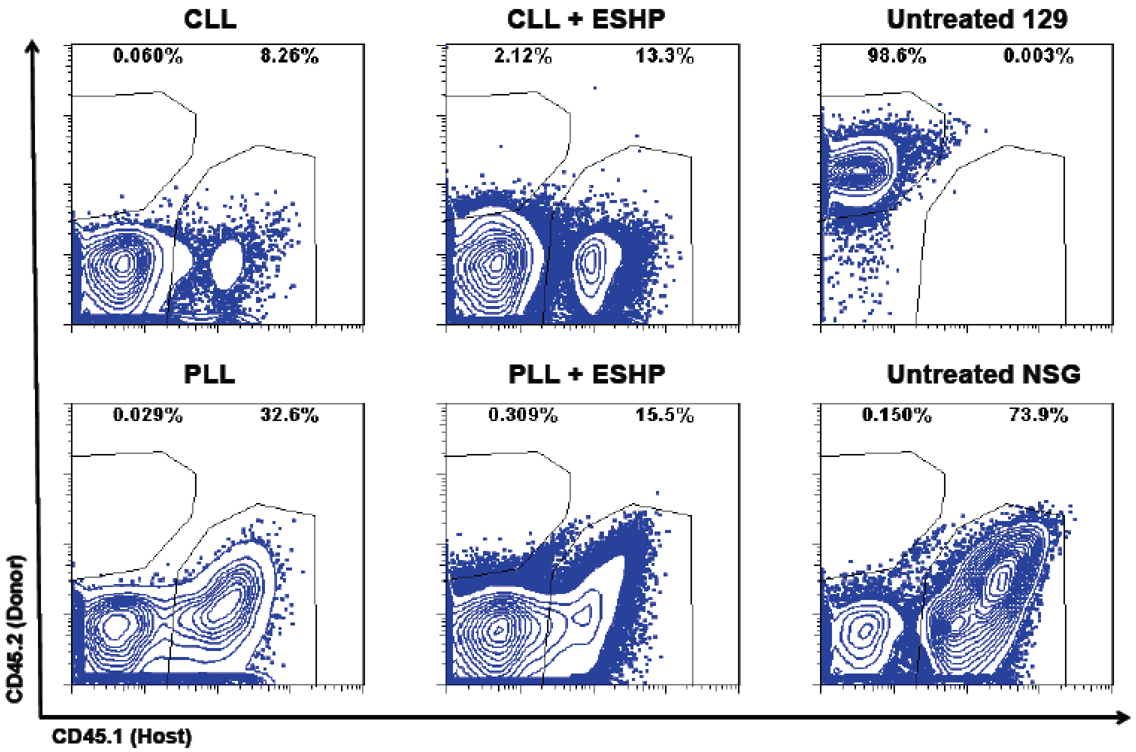
CLL
0.060%
8.26%
CLL + ESHP
2.12%
13.3%
Untreated 129
98.6%
0.003%
PLL
0.029%
32.6%
PLL + ESHP
0.309%
15.5%
Untreated NSG
0.150%
73.9%
CD45.2 (Donor)
CD45.1 (Host)

E

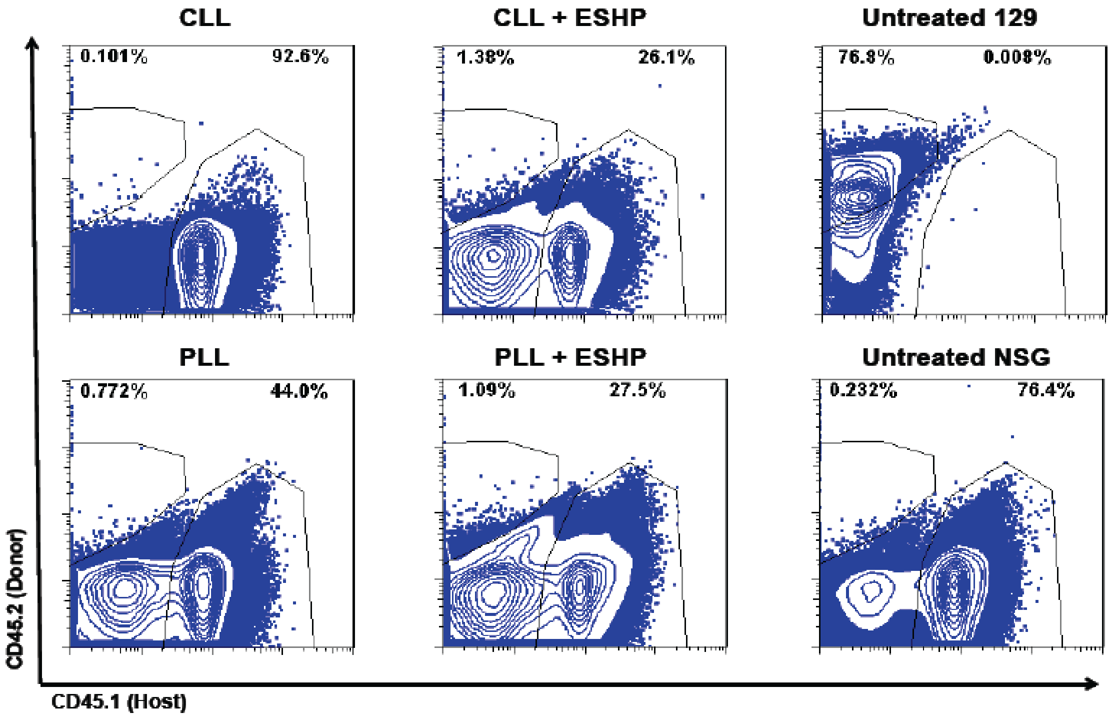
CLL
0.101%
92.6%
CLL + ESHP
1.38%
26.1%
Untreated 129
76.8%
0.008%
PLL
0.772%
44.0%
PLL + ESHP
1.09%
27.5%
Untreated NSG
0.232%
76.4%
CD45.2 (Donor)
CD45.1 (Host)

Supplementary Figure 6. Expression of macrophage inhibitory ligands CD47 and CD200 on ESHP. Isotype controls are shown in black-gray shaded regions and specific expression is shown in blue.

Figure S6

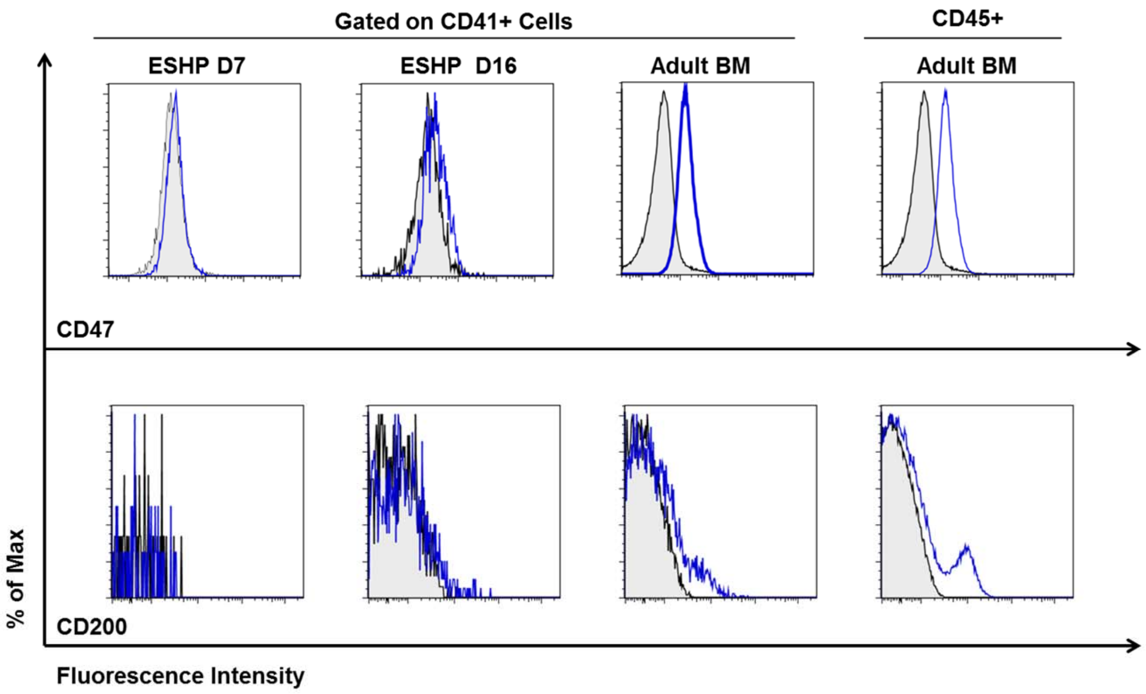
Gated on CD41+ Cells
CD45+
ESHP D7
ESHP D16
Adult BM
Adult BM
CD47
% of Max
CD200
Fluorescence Intensity
